# Exploring the Involvement of *ERAP1* in the Pathogenesis of Non-Infectious Uveitis: A Systematic Review

**DOI:** 10.3390/life16081340

**Published:** 2026-08-16

**Authors:** Ioana-Maria Rizea, Horia Tudor Stanca, Vasile Potop, Dana-Margareta-Cornelia Dăscălescu, Silvia Ioana Andrei, Ioana Teodora Tofolean, Alina Popa-Cherecheanu, Mihnea Munteanu, Marius Cherciu, Olivia-Mihaela Popa

**Affiliations:** 1 Doctoral School, “Carol Davila” University of Medicine and Pharmacy, 020021 Bucharest, Romania; ioana-maria.vlad@drd.umfcd.ro; 2 Clinical Department of Ophthalmology, “Carol Davila” University of Medicine and Pharmacy, 020021 Bucharest, Romania; dana.dascalescu@umfcd.ro (D.-M.-C.D.); ioanatofolean@gmail.com (I.T.T.); alina.cherecheanu@umfcd.ro (A.P.-C.); 3 Clinical Department of Ophthalmology, “Prof. Dr. Agrippa Ionescu” Emergency Hospital, 011356 Bucharest, Romania; 4 Clinical Department of Ophthalmology, “Prof. Dr. Mircea Olteanu” Clinical Institute for Ophthalmological Emergencies, 010464 Bucharest, Romania; 5 Faculty of Medicine, Friedrich-Alexander University Erlangen–Nürnberg, 91012 Erlangen, Germany; sandrei@thueringen-kliniken.de; 6 Department of Internal Medicine II, Thüringen Clinics “Georgius Agricola”, 07318 Saalfeld, Germany; 7 Clinical Department of Ophthalmology, University Emergency Hospital, 050098, Bucharest, Romania; 8 Clinical Department of Ophthalmology, Victor Babes University of Medicine and Pharmacy, 300041 Timisoara, Romania; mihneam1@gmail.com; 9 Department of Pathophysiology and Immunology, “Carol Davila” University of Medicine and Pharmacy, 020021 Bucharest, Romania; marius.cherciu@umfcd.ro (M.C.); olivia.popa@umfcd.ro (O.-M.P.)

**Keywords:** uveitis, endoplasmic reticulum aminopeptidase 1, *ERAP1*, human leukocyte antigen, HLA-B*27, major histocompatibility complex, polymorphism, SNP, haplotype, immunology

## Abstract

**Background**: Non-infectious uveitis (NIU) is an inflammatory ocular condition often associated with systemic immune-mediated disorders. The pathogenesis of NIU is complex and involves genes from the Human Leukocyte Antigen (HLA) class I system. Endoplasmic reticulum aminopeptidase 1 (ERAP1) cleaves antigenic peptides to an optimal length for loading and presentation on HLA I molecules. In this systematic review, we aimed to summarize the current knowledge regarding how susceptibility to developing NIU is linked to single-nucleotide polymorphisms (SNPs) in the *ERAP1* gene, a locus well known for its connection to autoinflammatory disorders. **Methods**: We performed a PRISMA protocol-guided systematic review in various databases. **Results**: Out of the 311 screened studies, only 12 met the inclusion criteria. Five of them are Genome-Wide Association Studies. We found relevant research for three types of NIU: acute anterior uveitis, Behçet’s uveitis, and birdshot chorioretinopathy. A frequently observed association between *ERAP1* SNPs and disease risk was noted when analyzing the HLA-carriers (B*27, B*51, A*29), which points toward an epistatic interaction between *ERAP1* and the MHC class I complex. **Conclusions**: Our findings suggest that *ERAP1* gene variation is understudied in relation to NIU. A significant amount of research has also concentrated on risk stratification, and reliable markers for patient selection are needed, especially in view of emerging anti-ERAP therapies.

## 1. Introduction

Uveitis is defined as a group of diseases characterized by inflammation of the middle layer of the eye, called the uvea, which consists of the iris, ciliary body, and choroid. It represents a major cause of vision loss worldwide. From an etiological standpoint, uveitis is classified into infectious and non-infectious (NIU) types. Non-infectious etiologies can account for up to 90% of uveitis cases, with variations according to the geographic location. More specifically, in developed countries, NIU ranges from 67% to 90%, whereas in developing countries, it barely exceeds the infectious causes. NIU affects mostly young people (children to middle-aged adults), more frequently women [1,2].

The American epidemiological studies report a prevalence of NIU around 121:100.000 people, while in the Asian population the prevalence ranges from 152 to 194 per 100.000 [3,4]. The incidence rate of NIU is about 25:100.000 individuals [4,5].

NIU has a significant impact on patients’ quality of life, due to the clinical signs and symptoms that include visual acuity loss, severe ocular pain, photophobia, and conjunctival hyperemia [6]. This affection has an increased rate of recurrence, as well as a high potential for ophthalmological complications such as band keratopathy, cataract, secondary glaucoma, macular edema, epiretinal membrane, or exudative retinal detachment [7].

The pathogenesis of NIU is complex and not fully understood, but it is thought to be caused by the interplay between environmental and genetic factors [8]. Studies involving NIU patients revealed genetic susceptibility for Human Leukocyte Antigen (HLA) genes (Table 1). HLA-A*29 reaches an impressive prevalence of up to 95.7% in patients with a type of NIU called Birdshot chorioretinopathy (BSCR) [9]. Therefore, BSCR is the prototype for major histocompatibility complex class I-opathies (MHC-I-opathies) [10]. Also, the association between HLA-B*27 and acute anterior uveitis (AAU) is well known, with approximately 50% of patients with AAU being HLA-B*27 positive [11]. However, the role of other markers in the pathogenesis of NIU, especially loci from outside the HLA group, still remains unclear [8,12,13]. Some of these studies have focused on investigating the role of endoplasmic reticulum aminopeptidases (ERAP) single-nucleotide polymorphisms (SNPs) in the susceptibility to develop various types of non-infectious uveitis [14].

Endoplasmic reticulum aminopeptidase 1 (ERAP1) is a multifunctional polymorphic enzyme included in the oxytocinase subfamily of M1 aminopeptidases [17]. Among the many functions of ERAP1, its role in regulating the human immune response is particularly important. The enzyme cleaves antigenic peptides (immunopeptidome) to an optimal length—from 10–16 amino acids (aa) to 8–9 aa, so that they can be loaded onto class I HLA molecules and then presented on the cell surface to CD8+ T cells [18,19,20]. This trimming function makes the ERAP1 enzyme a key determinant of the quantity and quality of the immunopeptidome repertoire. In vitro studies that have induced a decrease in ERAP1 expression showed that conventional peptide-MHC I complexes were lost from the cell surface and were replaced by structurally atypical, less stable, and frequently more immunogenic complexes [18,21,22].

A different role of ERAP1 in the immunological response is through its affinity for certain cytokine receptors: Interleukin-1 Receptor 2 (IL-1R2), Tumor Necrosis Factor Receptor 1 (TNF-R1), and Interleukin-6 Receptor alpha (IL-6Rα) [17]. Other possible roles of ERAP1 described in the literature include: inactivation of peptide hormones, blood pressure control by inhibiting angiotensin II and/or increasing bradykinin levels in the kidneys, angiogenesis modulation [23,24,25].

The human *ERAP1* gene is located on band 5q15 and comprises 20 exons and 19 introns according to the Genome Reference Consortium Human Build (GRCh38.p14) [26,27]. Exons 6 and 7 encode the zinc-binding domain responsible for the enzymatic activity of ERAP1 [28]. Through the phenomenon of alternative splicing at exon 19, the expression of *ERAP1* can result in two isoforms with distinct C-terminal ends. Isoform 1 or “a” is the longest one and contains 948 amino acids, of which the 1–939 aa are equivalent to isoform 2. The rest of isoform 1 (aa 940–948) are transcribed from exon 20. Isoform 2 or “b” contains 941 amino acids and is far more prevalent than isoform 1. Therefore, it was defined as the canonical sequence [28,29,30,31]. According to data from The National Center for Biotechnology Information (last updated on 5 August 2026), the *ERAP1* gene has 19 splicing variants [32].

The *ERAP1* gene shows the most significant expression in the small intestine, colon, and appendix, but no tissue specificity has been demonstrated, as the gene is ubiquitously expressed [33]. *ERAP1* is expressed codominantly, which implies that both the function of the protein and its involvement in various pathologies are determined by the allotypes present on both chromosomes [34].

The *ERAP1* gene is highly polymorphic within the human population, with over 40,000 single-nucleotide polymorphisms (SNPs) described (approximately 76% of the gene). Some of these SNPs have a significant impact on the enzymatic activity and/or the expression level [35,36,37].

Among the coding SNPs, rs30187 is the most studied within the *ERAP1* gene, along with rs27044 [38]. Both of them are missense SNPs, known for affecting the enzymatic activity of ERAP1 protein [39]. The first one encodes the substitution of lysine (K) in the 528 position with arginine (R) -Lys528Arg-, which converts the ERAP1 enzyme from the active form to an inactive state. The second one leads to the replacement of glutamine (Q) in position 730 with glutamic acid (E) (Gln730Glu), which alters the way the enzyme cleaves and processes peptides for presentation to the immune system [36,39,40]. Other common *ERAP1* SNPs and their mechanism of action, thus clinical significance, are illustrated in Supplementary Table S1 [41,42,43,44,45,46,47,48,49,50].

Haplotypes are distinct arrangements of SNPs that encode the allotypes—protein variations exhibiting differences in binding affinity and enzymatic activity [51,52]. Based on 9 tagging SNPs genotyped in the HapMap CEU, Ombrello et al. identified ten *ERAP1* haplotypes with frequencies > 1% in at least one population [42].

Subsequent studies have shown that these haplotypes are associated with different levels of ERAP1 enzymatic activity (increased, moderate, or decreased) and are linked to various disease susceptibilities (Supplementary Table S2) [20,42,53,54,55,56,57,58].

We performed a systematic review of the literature in order to provide an overview of *ERAP1* gene variation in relation to the risk of non-infectious uveitis. The results of our study allow us to identify knowledge gaps in this field and also future research perspectives.

## 2. Materials and Methods

To identify relevant studies, we conducted a comprehensive literature search on PubMed, Scopus, and the Clarivate Web of Science (WoS) Core Collection up to 19 June 2026. The search strategy was based on using the combination of the following key words: (ERAP1 OR “ERAP 1” OR ERAP-1 OR “Endoplasmic reticulum aminopeptidase 1” OR “Endoplasmic reticulum aminopeptidase1” OR “Endoplasmic reticulum aminopeptidase-1” OR “aminopeptidase regulator of TNFR1 shedding” OR ARTS-1 “ARTS 1” OR ARTS1 OR “adipocyte-derived leucine aminopeptidase” OR “adipocyte derived leucine aminopeptidase” OR “A-LAP”) AND (uveitis OR iridocyclitis OR panuveitis OR “Birdshot chorioretinopathy” OR Birdshot OR “Vogt-Koyanagi-Harada” OR “Vogt Koyanagi Harada” OR VKH OR “sympathetic ophthalmia”). In order to avoid limiting the diversity of scientific evidence and to ensure that certain valuable studies were not excluded based on the writing language or the year of the research, no search filters were used, thus reducing the risk of systematic errors.

In addition, to ensure comprehensive coverage of the literature, we conducted an additional search of the gray literature, including clinical trial registers (e.g., ClinicalTrials.gov, World Health Organization-International Clinical Trials Registry Platform), Bielefeld Academic Search Engine (BASE), Open Access Theses and Dissertations (OATD) platform and the Association for Research in Vision and Ophthalmology (ARVO)’s Journal-Investigative Ophthalmology and Visual Science (IOVS), to identify potential ongoing or unpublished clinical trials, doctoral theses or conference abstracts from other research that evaluates the role of *ERAP1* in susceptibility to develop uveitis that are not indexed in the mainstream databases.

The duplicate articles returned by the above-mentioned sources were removed using the Zotero software (version 9.0.5; Digital Scholar, Falls Church, VA, USA).

Two independent researchers (I.-M.R. and O.-M.P.) reviewed the titles and abstracts for screening criteria (Table 2). The selected articles were read in full to verify the inclusion criteria (Table 3).

**Figure 1:**
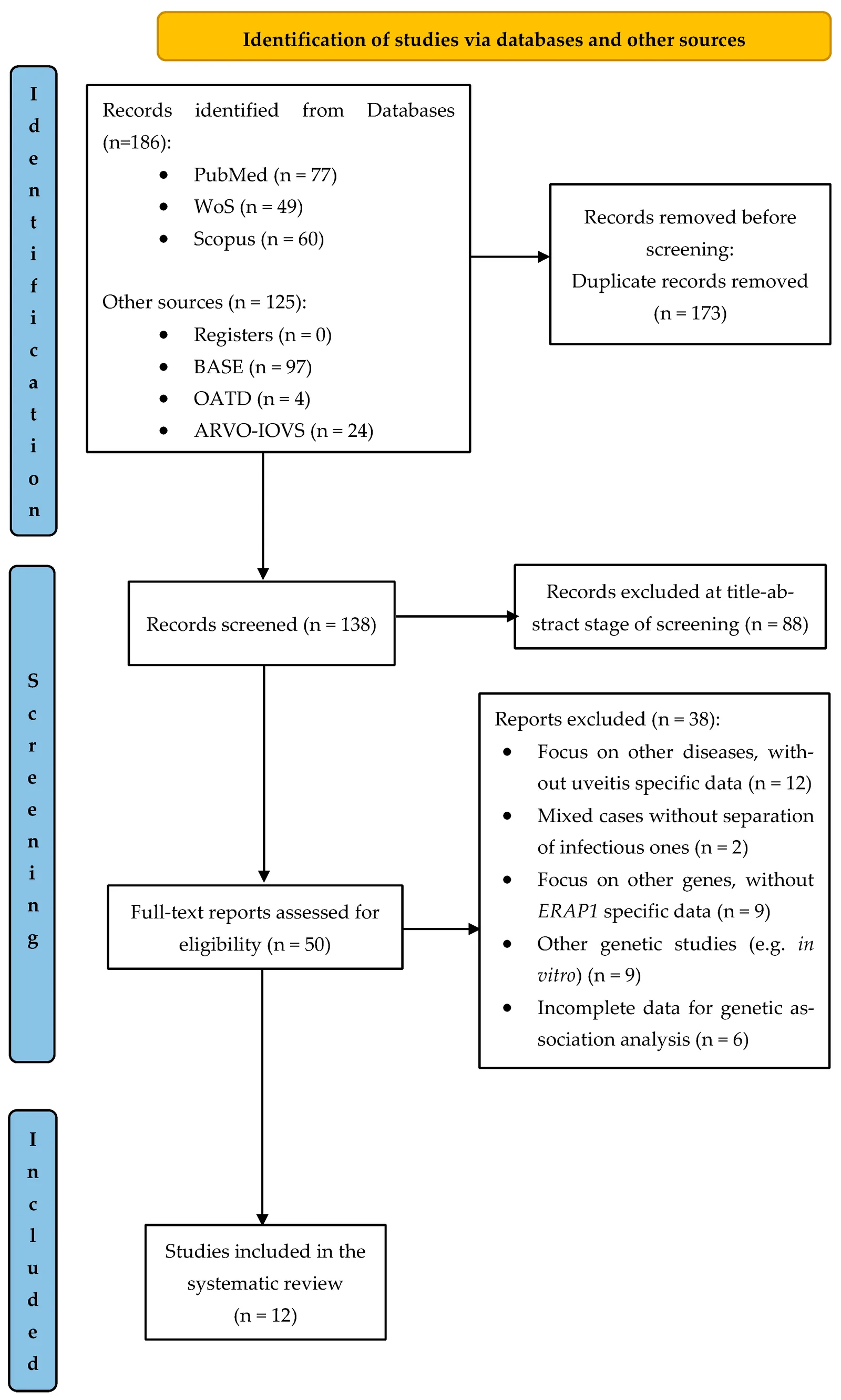
PRISMA flow diagram for *ERAP1* studies in non-infectious uveitis.

We followed the guidelines from the Preferred Reporting Items for Systematic reviews and Meta-Analyses (PRISMA) for displaying our results [59]. Thus, we designed the PRISMA diagram to summarize the search process and make it more straightforward to track the workflow (Figure 1, Supplementary Tables S3 and S4). For each eligible article, data were extracted regarding the author’s name, publication year, population (ethnicity), type of uveitis, number of patients and controls, *ERAP1* variants studied, and main results-*p*-value, Odds Ratio (OR), 95% confidence interval (95% CI).

**Table 3:** Eligibility criteria using Population–Exposure–Comparison–Outcome (PECO) concept described in the *Cochrane Handbook for Systematic Reviews of Interventions* [60].

PECO	Element	Characteristics
**P**	Population	Patients diagnosed with NIU (anterior, intermediate, posterior, or panuveitis).
**E**	Exposure	The presence of *ERAP1* genetic variants.
**C**	Comparison	Healthy controls and/or systemic disease without NIU.
**O**	Outcome	Susceptibility to NIU (genetic association with clinical phenotype or disease severity).

NIU: non-infectious uveitis.

## 3. Results

The preliminary systematic literature search identified a total of 311 records: 186 entries found in databases (PubMed, WoS, Scopus) and 125 returned from gray literature screening (ClinicalTrials.gov, World Health Organization-International Clinical Trials Registry Platform, BASE, OATD, ARVO-IOVS Journal). PRISMA diagram summarizes the selection process (Figure 1). Following the exclusion of 173 duplicate articles, inclusion and exclusion criteria were applied, which led to 12 research papers being included in our systematic review.

Among these eligible studies, five focused on analyzing the association between *ERAP1* gene variants and susceptibility to developing ankylosing spondylitis-associated anterior uveitis. The remaining seven studies were distributed as follows in terms of etiology: three of them investigated the relationship between *ERAP1* SNPs and the risk of birdshot chorioretinopathy (BSCR), while the other four investigations focused on the risk of Behçet’s disease-associated uveitis (BU).

A comprehensive overview of the characteristics of each study, such as sample sizes and specific *ERAP1* gene SNPs assessed, is detailed in Table 4, Table 5 and Table 6.

## 4. Discussion

Our review focused on *ERAP1* gene single-nucleotide polymorphisms as risk factors for the broad group of non-infectious uveitis. We identified a total of 12 studies published between 2013 and 2026 that met this goal.

### 4.1. ERAP1 and Anterior Uveitis Associated with Ankylosing Spondylitis

Ankylosing spondylitis (AS) is a chronic, inflammatory rheumatic disease that mainly involves the spinal column and pelvic area, resulting in restricted joint mobility and a decline in the overall quality of life. Besides the well-known association between AS and the presence of the HLA-B*27 antigen, new risk loci in the non-HLA region have been identified, including polymorphisms of *ERAP1* gene [67,68].

Acute anterior uveitis (AAU) is the most prevalent and clinically significant extra-articular manifestation of ankylosing spondylitis, with approximately one-quarter of AS patients developing AAU during their lifetime [69,70,71]. Thus, researchers have extended the new immunogenetic findings in the field of AS to uveitis associated with this condition, considering these genetic markers as possible independent risk factors for ocular inflammation [72].

There are two Genome-Wide Association Studies (GWAS) conducted by Robinson et al. in 2015 and 2016 on Caucasian populations that indicate a possible link between *ERAP1* and AAU associated with AS [61,62]. In the study from 2015, a suggestive association was found between the intronic *ERAP1* SNP rs2032890 and the risk of AAU in AS patients compared to AS cases without AAU, while the missense variant rs30187 failed to reach the GWAS threshold value for statistical significance. The association with rs2032890 remained significant in the analysis of HLA-B*27-positive subgroups (*p* = 1.6 × 10^−5^, OR: 1.26, 95% CI 1.13–1.40), but not in negative ones [61].

On the other hand, the study from 2016 demonstrated a significant risk for rs30187 in the analysis of AS with AAU compared to healthy controls, while the missense variant rs2287987 (Met349Val) showed a protective trend against the disease. The analysis of AS patients without AAU compared to AS with AAU showed a significant association for rs30187 for HLA-B*27 positive subgroups (*p* = 1.78 × 10^−3^; OR: 1.19; 95% CI 1.07–1.33) [62].

Another GWAS focused on the European population was coordinated by Huang et al. in 2020 [46]. They found a different AAU susceptibility locus than the previous studies, the risk allele A of the missense SNP rs27529 (Ser453Arg) being more frequent in AS patients with AAU than in the subgroup of AS without AAU. The genome-wide significance was reached for rs30187 in the subanalysis of B*27-positive AS-associated AAU cases compared to all AS patients without AAU (*p* = 1.4 × 10^−8^; OR: 1.25) [46].

The only cross-sectional cohort study included in the systematic review is the one made by Nossent et al. in 2016 [63]. They followed up a cohort of Caucasian subjects from Norway over an eight-year period. They found that the carriers of rs27044/rs30187 CT haplotype (the so-called loss-of-function haplotype of *ERAP1* gene) had a significantly lower risk of developing uveitis (Table 4) [63].

The *ERAP1* variants reported in the research of Su et al. (2018) [64] on an Asian cohort show a significant association with the risk of disease for Chinese AS patients with AAU for three SNPs (rs27037, rs30187, rs27434) at the allelic level (Table 4). The frequency of HLA-B*27 in patients was 82.92%, whereas no information was available for healthy controls. These associations lost significance after correction for multiple testing. In contrast with data reported for the Caucasian population, haplotype analysis did not show any associations. No differences were observed between AS with AAU versus AS without AAU for the investigated SNPs [64].

The results of these studies indicate that the association of *ERAP1* variants with the risk of AAU is dependent on the presence of the HLA-B*27 marker. The mechanism behind this association relies on the expression of different B*27 peptidomes driven by *ERAP1* haplotypes. Findings from AS-related research showed that cell lines with *ERAP1* Hap10 (which is a low-expression, low-activity haplotype associated with protection against AS) express a different B*27 peptidome compared with risk Hap2 by providing reduced trimming of peptides [20,73]. Moreover, studies from mouse models or human cell lines with reduced or lost ERAP1 expression show the presence of unusual peptide-MHC I complexes presented to the cell surface. They were unstable and highly immunogenic, while most conventional complexes disappeared [22]. Additional mechanisms involving other ERAP1 functions may also contribute to *ERAP1* involvement in AAU.

### 4.2. ERAP1 and Behçet’s Disease-Associated Uveitis

Behçet’s disease (BD) is a chronic recurrent inflammatory disorder that affects multiple organs, including the eyes. The disease was first described in 1937 by Professor Hulusi Behçet. The etiology of this illness is still incompletely understood, with most of the studies pointing towards a genetic origin that affects the immune system associated with environmental factors. The prevalence of the disease is higher in the Middle East and East Asia compared with other regions worldwide, making it known as “the silk road disease” [74,75]. The primary genetic association for BD is with HLA-B*51, the reported frequency being between 50 and 80% in BD patients from the above-mentioned endemic regions [76].

Some studies found that 90% of the patients with Behçet’s disease have ocular involvement. It can cause anterior, intermediate, posterior uveitis or even panuveitis, involving all the parts of the eye. As a recurrent disease, every reactivation represents a new risk of vision loss [75,77,78,79].

Kirino et al. conducted a Genome-Wide Association Study (GWAS) on Turkish patients with Behçet’s disease in 2013. They reported no significant differences between the allelic distribution within the uveitis subgroup versus healthy controls for three *ERAP1* SNPs: two missense variants, rs17482078 (Arg725Gln) and rs10050860 (Asp575Asn), and the intronic SNP rs2927615. However, when the recessive model was applied, the homozygous genotype of the risk alleles was prevalent in Behçet’s uveitis (BU) for all mentioned SNPs (Table 5), with an increased risk significance for rs17482078 in the meta-analysis conducted on BU patients from the discovery and replication cohorts from this study (*p* = 4.73 × 10^−11^; OR: 4.56; 95% CI 2.88–7.22). No analysis was performed for *ERAP1* SNPs in relation to HLA-B*51 for BU patients in this study [48].

Consistent with this result is the research conducted by Sousa et al. (2015) in an Iranian cohort. They found that *ERAP1* SNPs rs10050860 (Asp575Asn) and rs13154629 (intronic) were related to the risk of Behçet’s disease-associated uveitis using the recessive model. Moreover, they obtained a marginal association (*p* = 4.84 × 10^−2^; OR: 3.36; 95% CI 1.01–11.21) for the risk allele T of rs10050860, using the same recessive model, in a subgroup of BU patients carrying the minor allele A of rs76546355 (a proven marker for HLA–B*51 in Iranians) [49]. Three years later, a different study involving Iranian subjects (Mahmoudi et al., 2018) reported an increased risk of developing BU for *ERAP1* SNP rs30187. HLA-B*51 was present in 66% of BU patients. A marginal association (*p* = 0.057; OR: 1.74; 95% CI 0.98–3.11) was observed in this subgroup of B*51-positive uveitis cases for the risk allele T of rs30187, under the recessive model (TT vs. CT + CC), while the analysis in the whole BU cohort failed to reach statistical significance (Table 5) [65].

On the other hand, the effects of *ERAP1* SNPs in the Asian population with BU are contradictory. Zhang et al. found in their study from 2015 a significant association for the missense variant rs10050860 (Asp575Asn) with protection from developing uveitis in Chinese Han individuals with Behçet’s disease. The same effect has also been observed for the intronic variant rs1065407 (Table 5). There are no data regarding HLA-B*51 analysis in this study [57].

These observations highlight the need for further research for this type of NIU, with cohorts covering more populations and extended HLA typing along with *ERAP1* haplotype analysis.

### 4.3. ERAP1 and Birdshot Chorioretinopathy

Birdshot chorioretinopathy (BSCR) is a rare, chronic ocular disease that affects the posterior segment of the eye (choroid and retina). The clinical presentation is that of bilateral posterior uveitis. While the exact cause of this disease is not yet fully understood, it is presumed to be connected to an underlying autoimmune response [80,81,82]. The primary genetic risk factor for BSCR is HLA-A*29 with a frequency ≥90% in these patients. The approximate prevalence of BSCR is around 0.1–0.6:100,000 in the general population. It seems to have a predilection for European ancestry, HLA-A*29 being as well quite frequent in European descendants. Several studies have examined the role of *ERAP1* gene variants in this context [47,50,66,82].

The first association study between BSCR and *ERAP1* was performed by Kuiper et al. in 2018, on both Dutch and Spanish cohorts. Their findings on haplotype association revealed different results: for the Dutch population, haplotypes Hap1 and Hap2 were protective, and a protective tendency was observed for Hap6, while Hap10 seemed to be a risk factor in both Dutch and Spanish study groups (Table 6). After correction, the only preserved association was the risk effect of Hap10 in the Dutch cohort. This association was also observed in the combined analysis of the two populations: *p* = 2.88 × 10^−6^; OR: 1.99; 95% CI 1.49–2.65. The same discrepancy between the two ethnic groups was also noticed at the individual SNP level regarding the association with BSCR. Several nominally significant data resulted from the allelic analysis (Table 6). After correction, all the associations lost significance in the Spanish cohort, while in the Dutch population, the missense *ERAP1* variant rs30187 was still associated with protection against BSCR, and the missense SNPs rs2287987 (Met349Val), rs10050860 (Asp575Asn), and rs17482078 (Arg725Gln) remained significant risk markers for BSCR. For the analysis of *ERAP1* variants in HLA-A*29-positive subjects, both populations were used. A total of 129 BSCR patients and 439 controls were included. A significant association with the risk of disease was observed for the rs2287987-CC genotype in combination with the rs10044354-TT genotype from another aminopeptidase gene, *LNPEP (Leucyl and Cystinyl Aminopeptidase)* (*p* = 2.4 × 10^−7^; OR: 41.4; 95% CI 10.08–170.08) [50].

Another study focused on the correlation between *ERAP1* variability and BSCR is the GWAS conducted by Gelfman S. et al. in a French cohort in which the HLA-A*29 marker was present in both patients and healthy controls. Significant findings were reported regarding the association between the minor allele of the *ERAP1* intronic SNP-rs27432, and the risk of BSCR (*p* = 6.6 × 10^−7^; OR: 2.58). This result was confirmed by the meta-analysis of the French cohort combined with the Dutch and Spanish cohorts previously reported (*p* = 4.07 × 10^−10^; OR: 2.46, 95% CI 1.85–3.26). Moreover, they highlighted the additive effect for BSCR risk in subjects with the risk allele G of rs27432, HLA-A*29, and another allele from the AW*19 group. Haplotype-level analysis suggests that *ERAP1* could modulate the susceptibility to developing BSCR: Hap1 and Hap2 (driving increased enzymatic activity) showed a suggestive protective role against BSCR, while Hap10 (reduced ERAP1 activity) was more frequently found in patients than in controls, but without reaching the GWAS significance. Also, they found that both *ERAP1* and *ERAP2* increase the likelihood of BSCR when testing the haplotype *ERAP1*rs27432/*ERAP2*rs10044354 G/T. Thus, they pointed out that elevated ERAP2 levels and decreased ERAP1 activity are connected to an increased risk of BSCR compared to each of these factors alone [47].

A more recent study on the French population with BSCR was published by Loeliger, Gelfman et al. in 2026 [66]. They used the same control group as in the GWAS, while the patient group was increased with 120 subjects, all of whom were HLA-A*29 positive. The same above-mentioned pattern of low ERAP1 and high ERAP2 activity was noticed in the BSCR cohort. The haplotypic investigation revealed a substantially protective effect against developing the disease for Hap1 and Hap2, while Hap5 and Hap10 posed a considerable risk of BSCR. The nominal risk haplotype Hap8 reached only a marginal significance after multiple testing (Table 6). In addition to the previous studies, they investigated the role of these genes in the severity and visual acuity progression in BSCR, but without significant results [66].

These findings point to a certain pathogenic role of *ERAP1* together with *ERAP2* in BSCR etiology, in the presence of the HLA-A*29 antigen. The mechanism behind this association implicates a modified immunopeptidome with low affinity to A*29, which causes a reactive response of the CD8+ T lymphocytes. In vitro studies that investigated the effect of a highly active ERAP1 in the absence of ERAP2 enzymatic activity model (in opposition to the low ERAP1 and high ERAP2 model associated with BSCR) showed that the generated peptides had an increased affinity for A*29:02 [20,83]. Also, infiltrated CD8+ and CD4+ T cells found in the vitreous fluid samples of BSCR patients support this hypothesis [84]. More studies are needed to clarify the pathological implication of *ERAP1* in BSCR.

Regarding ERAP1 modulation for future therapies, in the last decade, research efforts were made in order to develop ERAP1 inhibitors, as ERAP1 and ERAP2 are considered promising targets for the treatment of various cancers and inflammatory diseases, especially those linked to the MHC system [85,86]. There is currently an ongoing phase I/II clinical trial, the ERAP Mediated Immunopeptidome Targeting Trial–1 (EMITT-1), that tests the effect of an ERAP1 inhibitor (GRWD5769) in locally advanced or metastatic solid tumors [87,88]. While there are several pharmacological inhibitors and enhancers that have been evaluated in vitro for their impact on antigen presentation in inflammatory diseases linked to the MHC system, such as ankylosing spondylitis, comprehensive in vivo assessment is still lacking [89]. Unlike cancer immunotherapy, where ERAP1 inhibition shows strong promise, autoimmune ocular diseases present a particular risk profile [56,86]. Because ERAP1 activity is HLA-allele-dependent (for example, haplotypes that are protective in HLA-B*27, but pathogenic in HLA-B*51 and HLA-A*29, as we saw in the earlier discussed studies), non-selective modulation comes with the potential risk of exacerbation, rather than attenuation of the intraocular inflammation [56]. The use of these agents in NIU and/or other inflammatory diseases requires a significant amount of research, with a focus also on risk stratification of patients, safety assessment, and reliable markers for patient selection.

Based on the results of the present systematic review, we can state that *ERAP1* gene variation is insufficiently studied in non-infectious uveitis. While our search strategy broadly targeted the entire spectrum of NIU, the current literature is exclusively limited to three HLA class I-associated uveitides: acute anterior uveitis associated with ankylosing spondylitis, Behçet’s uveitis and birdshot chorioretinopathy. This highlights a significant gap in the literature and underscores the need for future research to expand these investigations across a wider range of non-infectious uveitis.

## 5. Conclusions

For all the above-mentioned non-infectious uveitis clusters, a frequently observed association between *ERAP1* SNPs and disease risk was noted in the subanalysis of the subjects carrying a specific HLA marker (B*27, B*51, A*29). These results reinforce the epistatic interaction between HLA-system and *ERAP1* gene noted in previous studies on immune-mediated diseases and highlight the fact that such genetic association research should also include a stratification analysis based on the uveitis-specific HLA. Otherwise, the true effect of *ERAP1* genetic variation will be diluted when assessing the NIU cohort as a whole, underestimating its actual impact in carriers of a particular HLA molecule.

The ethnic diversity seems to have a strong reflection in the genetic profile of these HLA class I-associated NIU. These epidemiological discrepancies emphasize the fact that, in order to analyze the role of the ERAP1-HLA axis in the pathogenesis of NIU, we must view these immunological mechanisms through a population-specific lens.

## Figures and Tables

**Table 1:** Non-infectious uveitis (NIU) association with HLA [8,9,11,12,13,15,16].

Type of Uveitis	HLA Type
Acute anterior uveitis associated with spondyloarthritis	HLA-B*27
Behçet’s disease	HLA-B*51, HLA-B*5
Birdshot chorioretinopathy	HLA-A*29
Vogt–Koyanagi–Harada syndrome	HLA-DR*4
Sympathetic ophthalmia	HLA-DR*4

**Table 2:** Screening criteria.

Exclusion Criteria	Inclusion Criteria
-Genetic association studies between *ERAP1* and other conditions besides NIU; -Studies in which uveitis cases were identified solely through administrative/ICD diagnostic codes, without clinical characterization (to allow exclusion of infectious etiology); -Studies evaluating other genetic loci rather than *ERAP1*; -Descriptive studies (Case Reports, Case Series), Review or Editorial type of papers; -In vitro/Animal Model Studies; -Other genetic research than genetic association studies; -Lack of results from the genetic association analysis.	-Patients diagnosed with NIU; -*ERAP1* SNPs genetic association studies; -Observational studies: Case–control, cohort, cross-sectional, Genome-Wide Association Studies.

NIU: non-infectious uveitis; ICD: International Classification of Diseases; SNPs: single-nucleotide polymorphisms.

**Table 4:** Studies regarding *ERAP1* implication in the pathogenesis of ankylosing spondylitis-associated non-infectious uveitis based on allelic model or haplotype analysis [46,61,62,63,64].

Authors	Study Design	Population (Ethnicity)	Cases/Controls	Type of Uveitis (Etiology)	Investigated SNPs or Haplotypes	Results		Genetic Association
						*p*	OR (95% CI)	
Robinson P.C. et al., 2015 [61]	GWAS	Caucasian (British, Australian, American, Canadian)	1422/2339	AAU (AS)	rs2032890 rs30187	9.0 × 10^−6^ 0.01	1.3 (1.2–1.5) 0.87 (0.78–0.97)	Suggestive association with the risk ** Lack of association for GWAS **
Robinson P.C. et al., 2016 [62]	GWAS	Caucasian (British, Australian, New Zealand)	1409/20,164	AAU (AS)	rs30187 rs2287987	2.80 × 10^−21^ 7.8 × 10^−7^	1.46 (1.35–1.58) 0.76 (0.68–0.85)	Risk ** Suggestive association for protection **
Nossent J.C. et al., 2016 [63]	Cross-sectional, cohort	Caucasian (Norwegians)	32/302 ^‡^	Uveitis (AS)	rs27044/rs30187:			
					-C/C	ns	n/a	Lack of association
					-G/T	ns	n/a	Lack of association
					-C/T	0.024	0.31 (0.11–0.86)	**Protection**
Su W. et al., 2018 [64]	Case–control	Asian (Chinese)	884/1727	AAU (AS)	rs27037	0.02	1.14 (1.01–1.29)	**Risk ^†^**
					rs27434	0.02	1.13 (1.00–1.28)	**Risk ^†^**
					rs27980	ns	1.10 (0.97–1.23)	Lack of association
					rs30187	0.03	1.13 (1.00–1.27)	**Risk ^†^**
					rs27582	ns	0.94 (0.84–1.06)	Lack of association
					rs1065407	ns	1.08 (0.84–1.40)	Lack of association
					rs2032890	ns	0.98 (0.75–1.27)	Lack of association
					rs27044	ns	1.08 (0.96–1.21)	Lack of association
					rs27980/rs27582:			
					-TG	ns	n/a	Lack of association
					-GA	ns	n/a	Lack of association
					-TA	ns	n/a	Lack of association
					rs27980/rs27582:			
					-TG	ns	n/a	Lack of association
					-GA	ns	n/a	Lack of association
					-TA	ns	n/a	Lack of association
					rs30187/rs27434:			
					-TA	ns	n/a	Lack of association
					-CG	ns	n/a	Lack of association
					-CA	ns	n/a	Lack of association
					rs2549782/rs2248374:			
					-TG	ns	n/a	Lack of association
					-GA	ns	n/a	Lack of association
Huang X.F. et al., 2020 [46]	GWAS	Caucasian (British, Australian, American, French)	2752/3836	AAU (AS)	rs27529	2.19 ×10^−7^	1.22 (1.13–1.31)	Suggestive association with the risk **
					rs39841	7.91 × 10^−7^	n/a	Suggestive association **
					rs1057569	4.29 × 10^−4^	n/a	Lack of association for GWAS **

AAU (AS): Acute anterior uveitis associated with ankylosing spondylitis; SNPs: single-nucleotide polymorphisms; GWAS: Genome-Wide Association Study; †: Loss of significance after correction; ‡ AS without uveitis; ns = non-significant, n/a = not available; ** Statistical significance for GWAS: *p* < 5×10^−8^: genome-wide significance; 5 × 10^−8^ ≤ *p* < 1 × 10^−4^: suggestive for GWAS. For candidate-gene studies, significance refers to the nominal threshold reported by the authors (*p* < 0.05), with correction for multiple testing indicated where applied.

**Table 5:** Studies regarding *ERAP1* implication in the pathogenesis of Behçet’s uveitis based on the allelic or recessive model [48,49,57,65].

Authors	Study Design	Population (Ethnicity)	Cases/Controls	Type of Uveitis (Etiology)	Investigated SNPs or Haplotypes	Results		Genetic Association
						*p*	OR (95% CI)	
Kirino Y. et al., 2013 [48]	GWAS and Meta-Analysis	Asian (Turkish)	435/1278	BU	rs2927615	1.02 × 10^−7 §^	n/a ^§^	**Suggestive association with the risk** ** ^§^
					rs17482078	2.03 × 10^−8 §^	4.21 (2.45–7.23) ^§^	**Risk** ** ^§^
					rs10050860	n/a ^§^	n/a ^§^	**Risk** ** ^§^
Zhang L. et al., 2015 [57]	Case–control	Asian (Chinese)	930/1704	BU	rs1065407	4.03 × 10^−9^	0.51 (0.41–0.64)	**Protection** ^¶^
					rs10050860	4.41 × 10^−7^	0.54 (0.43–0.69)	**Protection** ^¶^
					rs27044	ns	n/a	Lack of association
					rs149481	ns	n/a	Lack of association
					rs27038	ns	n/a	Lack of association
					rs27980	ns	n/a	Lack of association
					rs13167972	ns	n/a	Lack of association
					rs7711564	ns	n/a	Lack of association
Sousa I. et al., 2015 [49]	Case–control and Meta-analysis	Asian (Iranian)	550/828	BU	rs10050860 rs13154629	1.25 × 10^−3 §^ 2.37 × 10^−3 §^	3.15 (2.41–3.88) ^§^ 2.88 (2.17–3.59) ^§^	**Risk ^¶^** ^§^ **Risk ^¶^** ^§^
Mahmoudi M. et al., 2018 [65]	Case–control	Asian (Iranian)	498/776	BU	rs30187	0.89 ^§^	0.98 (0.71–1.34) ^§^	Lack of association ^§^

GWAS: Genome-Wide Association Study; BU: Behçet’s uveitis; SNPs: single-nucleotide polymorphisms; ns = non-significant; n/a = not available; §: recessive model; ¶: still significant after correction; ** Statistical significance for GWAS: *p* < 5 × 10^−8^: genome-wide significance; 5 × 10^−8^ ≤ *p* < 1 × 10^−4^: suggestive for GWAS. For candidate-gene studies, significance refers to the nominal threshold reported by the authors (*p* < 0.05), with correction for multiple testing indicated where applied.

**Table 6:** Studies regarding *ERAP1* implication in the pathogenesis of birdshot chorioretinopathy based on allelic model and haplotype analysis [47,50,66].

Authors	Study Design	Population (Ethnicity)	Cases/Controls	Type of Uveitis (Etiology)	Investigated SNPs or Haplotypes	Results		Genetic Association
						*p*	OR (95% CI)	
Kuiper J.J.W. et al., 2018 [50]	Case–control and Meta-analysis	Caucasian: Dutch Spanish *	84/890 46/2103 *	BSCR	rs30187	1.20 × 10^−5^	0.37 (0.24–0.58)	**Protection** ^¶^
						0.31 *	0.80 (0.52–1.23) *	Lack of association *
					rs27044	5.84 × 10^−4^	0.45 (0.29–0.71)	**Protection** ^†^
						0.10 *	0.67 (0.42–1.08) *	Lack of association *
					rs26653	1.87 × 10^−3^	0.50 (0.33–0.78)	**Protection** ^†^
						0.2 *	0.74 (0.46–1.18) *	Lack of association*
					rs72773968	0.03	0.48 (0.24–0.94)	**Protection** ^†^
						0.7 *	0.90 (0.50–1.61) *	Lack of association *
					rs2287987	1.49 × 10^−5^	2.25 (1.56–3.24)	**Risk** ^¶^
						0.02 *	1.70 (1.09–2.67) *	Risk * ^†^
					rs10050860	1.51 × 10^−5^	2.24 (1.56–3.24)	**Risk** ^¶^
						0.02 *	1.70 (1.08–2.67) *	Risk * ^†^
					rs17482078	3.53 × 10^−5^	2.20 (1.51–3.20)	**Risk** ^¶^
						0.01 *	1.73 (1.10–2.71) *	Risk * ^†^
					rs1057569	3.25 × 10^−3^	1.69 (1.19–2.40)	**Risk** ^†^
						0.15 *	1.37 (0.89–2.11) *	Lack of association *
					rs27895	0.82	0.92 (0.47–1.81)	Lack of association
						0.75 *	0.86 (0.35–2.11) *	Lack of association *
					rs3734016	0.35	1.42 (0.68–2.95)	Lack of association
						0.31 *	1.69 (0.62–4.58) *	Lack of association *
					rs26618	0.16	1.32 (0.90–1.92)	Lack of association
						0.53 *	0.85 (0.51–1.42) *	Lack of association *
					Hap1	0.032	0.48 (n/a)	**Protection** ^†^
						0.74 *	0.91 * (n/a)	Lack of association *
					Hap2	6.4 × 10^−3^	0.42 (n/a)	**Protection** ^†^
						0.09 *	0.56 * (n/a)	Lack of association *
					Hap3	0.09	0.46 (n/a)	Lack of association
						0.31 *	1.41 * (n/a)	Lack of association *
					Hap5	0.83	0.93 (n/a)	Lack of association
						0.47 *	0.69 * (n/a)	Lack of association *
					Hap6	0.056	0.46 (n/a)	**Marginal association. Protection tendency**
						0.52 *	0.76 * (n/a)	Lack of association *
					Hap7	0.35	1.41 (n/a)	Lack of association
						0.27 *	1.76 * (n/a)	Lack of association *
					Hap8	0.15	1.32 (n/a)	Lack of association
						0.52 *	0.85 * (n/a)	Lack of association *
					Hap10	3.45 × 10^−5^	2.20 (n/a)	**Risk** ^¶^
						0.01 *	1.71 * (n/a)	Risk * ^†^
Gelfman S. et al., 2021 [47]	GWAS and Meta-analysis	Caucasian (French)	286/108	BSCR	rs27432	6.6 × 10^−7^	2.58 (1.78–3.76)	Suggestive association with the risk **
					Hap1	6.7 × 10^−6^	0.41 (n/a)	**Suggestive association with protection** **
					Hap2	6.7 × 10^−6^	0.41 (n/a)	**Suggestive association with protection** **
					Hap3	0.11	1.32 (n/a)	Lack of association for GWAS **
					Hap5	0.11	1.32 (n/a)	Lack of association for GWAS **
					Hap6	0.11	1.32 (n/a)	Lack of association for GWAS **
					Hap7	0.11	1.32 (n/a)	Lack of association for GWAS **
					Hap8	0.11	1.32 (n/a)	Lack of association for GWAS **
					Hap10	8.0 × 10^−3^	1.78 (n/a)	
Loeliger J. et al., 2026 [66]	Case–control	Caucasian (French)	406/106	BSCR	Hap1	0.0001	0.44 (n/a)	**Protection** ^¶^
					Hap2	0.0005	0.44 (n/a)	**Protection** ^¶^
					Hap3	0.41	0.75 (n/a)	Lack of association
					Hap4	0.60	0.39 (n/a)	Lack of association
					Hap5	0.01	2.63 (n/a)	**Risk** ^¶^
					Hap6	0.56	0.84 (n/a)	Lack of association
					Hap7	0.19	0.61 (n/a)	Lack of association
					Hap8	0.03	1.55 (n/a)	**Risk ^†^**
					Hap10	0.001	1.89 (n/a)	**Risk ^¶^**
					Hap2.1	n/a	n/a	Lack of association
					Hap10.1	1	0.78	Lack of association

GWAS: Genome-Wide Association Study; SNPs: single-nucleotide polymorphisms; BSCR: birdshot chorioretinopathy; n/a = not available; * results from Spanish cohort; ¶: still significant after correction; †: Loss of significance after correction. ** Statistical significance for GWAS: *p* < 5 × 10^−8^: genome-wide significance; 5 × 10^−8^ ≤ *p* < 1 × 10^−4^: suggestive for GWAS. For candidate-gene studies, significance refers to the nominal threshold reported by the authors (*p* < 0.05), with correction for multiple testing indicated where applied.

## Data Availability

No new data were created or analyzed in this study. Data sharing is not applicable to this article.

## Supplementary Materials

The following supporting information can be downloaded at: https://www.mdpi.com/article/10.3390/life16081340/s1, Supplementary Table S1: Frequent single-nucleotide polymorphisms of *ERAP1* gene in the human population investigated in NIU studies—genetic characteristics (data from dbSNP-the ALFA project) [41,42,43,44,45,46,47,48,49,50]; Supplementary Table S2: The ten most common *ERAP1* haplotypes in the human population-structural and functional characteristics [42,53,54,55,56,57,58]; Supplementary Table S3: PRISMA 2020 for Abstract Checklist [59]; Supplementary Table S4: PRISMA 2020 Checklist [59].

## Abbreviations

The following abbreviations are used in this manuscript:

95% CI	95% Confidence interval
aa	Amino acid
AAU	Acute anterior uveitis
A-LAP	Adipocyte-derived leucine aminopeptidase
ARTS	Adipocyte-derived leucine aminopeptidase
ARVO	Association for Research in Vision and Ophthalmology
AS	Ankylosing spondylitis
BASE	Bielefeld Academic Search Engine
BD	Behçet’s disease
BSCR	Birdshot chorioretinopathy
BU	Behçet’s uveitis
CC	Cytosine–Cytosine
CD8+	Cluster of differentiation 8 positive
CT	Cytosine-Thymine
C-terminal	Carboxyl-terminal
EMITT-1	ERAP Mediated Immunopeptidome Targeting Trial–1
ERAP	Endoplasmic reticulum aminopeptidase
*ERAP1*	Endoplasmic reticulum aminopeptidase 1
GRCh38.p14	Genome Reference Consortium Human Build 38 patch release 14th
GWAS	Genome-Wide Association Studies
Hap	Haplotype
HLA	Human leukocyte antigen
ICD	International Classification of Diseases
IL-1R2	Interleukin-1 Receptor 2
IL-6	Interleukin-6
IL-6Rα	Interleukin-6 Receptor alpha
IOVS	Investigative Ophthalmology and Visual Science
LNPEP	Leucyl and Cystinyl Aminopeptidase
MHC	Major histocompatibility complex
NIU	Non-infectious uveitis
OATD	Open Access Theses and Dissertations
OR	Odds Ratio
PECO	Population–Exposure–Comparison–Outcome
PRISMA	Preferred Reporting Items for Systematic reviews and Meta-Analyses
SNP	Single-nucleotide polymorphism
TNF-R1	Tumor Necrosis Factor Receptor 1
VKH	Vogt–Koyanagi–Harada
WoS	Web of Science
